# Land-use influences the distribution and activity of high affinity CO-oxidizing bacteria associated to type I-*coxL* genotype in soil

**DOI:** 10.3389/fmicb.2014.00271

**Published:** 2014-06-12

**Authors:** Liliana Quiza, Isabelle Lalonde, Claude Guertin, Philippe Constant

**Affiliations:** Institut National de la Recherche Scientifique-Institut Armand-FrappierLaval, QC, Canada

**Keywords:** trace gas, soil uptake, atmosphere, global change, gas exchanges

## Abstract

Soil carboxydovore bacteria are the biological sink of atmospheric carbon monoxide (CO). The initial oxidation of CO is catalyzed by a CO-dehydrogenase (CODH), and the gene *coxL* encodes the large subunit of the enzyme. Only a few carboxydovore isolates were shown to oxidize atmospheric CO and little is known about the potential impact of global change on the ecophysiology of this functional group. The main objective of this study was to assess the impact of land-use and soil properties on *coxL* gene diversity and identify molecular indicators for the soil uptake of atmospheric CO. Soil samples were collected in three neighboring sites encompassing different land-use types, namely deciduous forest, larch plantation and maize field. CO uptake activity was related to total carbon and nitrogen content in soil, with the highest activity observed in deciduous forest. An extensive *coxL* database was assembled to optimize a PCR detection assay targeting sequences belonging to functional type I-CODH and hypothetical type II-CODH. Fully replicated *coxL* gene libraries unveiled a unique molecular signature in deciduous forest soil, with enrichment of type I sequences. Genetic profiles of larch and maize monocultures were not statistically different and showed higher level of *coxL* gene richness than deciduous forest. Soil water content and CO uptake activity explained 38% of the variation of *coxL* gene profiles in a canonical ordination analysis, leading to the identification of sequences belonging to the δ-Proteobacteria cluster as indicator for high affinity CO uptake activity. Enrichment of type I and δ-Proteobacteria *coxL* sequences in deciduous forest were confirmed by qPCR in an independent soil survey. CO uptake activity in model carboxydovore bacteria suggested that a significant fraction of detected putative high affinity CO oxidizers were active in soil. Land-use was a driving force separating *coxL* diversity in deciduous forest from monocultures.

## Introduction

Carbon monoxide (CO) is present at the trace level in the atmosphere, with typical background levels ranging from 1 ppmv in urban areas to 0.1 ppmv in remote locations (Novelli et al., 1998; Chan et al., 2002). A combination of modeling approaches attributed biogenic hydrocarbons and methane oxidation, biomass burning and fossil fuel utilization as the main sources of CO in the atmosphere, representing 2500 Tg CO year^−1^ global emissions (Holloway and Levy, 2000). This trace gas has a relatively short atmospheric lifetime of 0.4–2 months, owing to its strong reactivity toward hydroxyl radicals (OH·), the cleansing molecules in the atmosphere. Because of this OH·-mediated reaction, CO indirectly influences the distribution of methane, and thus is considered as an indirect greenhouse gas (Daniel and Solomon, 1998). The uptake of atmospheric CO, catalyzed by specialized microorganisms thriving in oxic soil, is the most uncertain term of CO budget, representing about 15% of the global loss of this trace gas in the atmosphere. Despite the fact that industrialization has increased CO global emissions in the last century (Assonov et al., 2007), current CO concentrations are relatively stable in the atmosphere. Long-term time series analysis unveiled slight but significant decreasing trends of CO concentration in response to reduced industrial and motor vehicle emissions, disturbed by episodic CO pulses originating from forest fires (Novelli et al., 2003; Chevalier et al., 2008). This delicate balance in the atmospheric burden of CO largely relies on microbiological and chemical processes responsible for the abatement of global emissions. Resilience, resistance, or vulnerability of the biological sink of CO to global change, including changes in land-use and climate, must be assessed to predict the fate of atmospheric CO budget. Identification and characterization of soil CO-oxidizing bacteria are mandatory to achieve this challenging task.

In general, CO is a highly toxic gas due to its high affinity to metal-containing enzymes involved in respiratory chains. Sophisticated CO-insensitive metabolisms have evolved in bacteria adapted to exploit this trace gas distributed ubiquitously in the environment. CO-oxidizing bacteria possess a CO-dehydrogenase (CODH) catalyzing the reaction: CO + H_2_O → CO_2_ + 2H^+^ + 2e^−^. The enzyme is a member of the molybdenum-containing hydroxylases comprising aldehyde oxidoreductase and xanthine dehydrogenase participating in purine nucleotide metabolism (Hille, 2005). In aerobic CO-oxidizing bacteria, CODH is a dimer of heterotrimers consisting of the small (CoxS), medium (CoxM), and large (CoxL) subunits (Dobbek et al., 2002). The active site embedded in the large subunit comprises a dinuclear molybdenum and copper heterometal cluster unique to CODH. Two physiological groups of aerobic CO-oxidizing bacteria have been identified. Carboxydotrophic bacteria are generally facultative chemolithoautotrophs, using CO as a sole carbon and energy source if organic substrates are growth limiting (Mörsdorf et al., 1992). These bacteria have a low affinity toward CO and are incapable of scavenging atmospheric CO (Conrad et al., 1981). In contrast, carboxydovore bacteria cannot grow in presence of elevated level of CO. These bacteria exhibit a versatile mixotrophic metabolism, allowing them to grow on mixtures of inorganic and organic substrates (King and Weber, 2007). In soil, carboxydovore bacteria scavenge atmospheric CO and take advantage of CO diffusing in soil from nitrogen-fixing nodules as well as chemical degradation of organic matter.

A few high affinity carboxydovore bacteria have been isolated so far and very little is known about the environmental control on their distribution and activity (King and Weber, 2007). Phylogenetic analysis of *coxL* gene sequences revealed the occurrence of two different groups of CODH, namely the functional type I-CODH and the hypothetical type II-CODH. Type I-CODH are the most extensively studied and are found in the model carboxydotrophic bacterium *Oligotropha carboxidovorans* as well as carboxidovores such as *Stappia* and *Mycobacterium* isolates demonstrating the ability to scavenge atmospheric CO (King, 2003a,b; Weber and King, 2007). Comparatively less is known about the second group, since the occurrence of a functional type II-CODH never has been reported. Distribution of type I- and type II-*coxL* sequence has been investigated in the environment. Although both phylogenetic groups are ubiquitous in soil, the environmental control on their distribution remains to be elucidated. The objective of this study is to assess the impact of land-use on carboxydovore activity and diversity. We tested the hypothesis that adjacent sites encompassing different land-use types harbor distinct CO-oxidizing bacterial community structure and density, resulting in a site-specific CO uptake activity and *coxL* diversity profile. Soil physicochemical parameters, known to vary within the three ecosystems, such as carbon content and pH, were expected to explain the spatial distribution of this functional group in soil due to the importance of these factors in shaping soil microbial communities structure (Vasileiadis et al., 2013).

## Materials and methods

### Sites and sampling

Soil samples were collected at the Verchères Arboretum in St. Amable, (QC, Canada), located about 40 km from Montreal on the south shore of the St. Lawrence River (45°67′N; 73°32′W). The landscape of the sampling site is a mosaic encompassing a broad range of ecosystem types arranged over a relatively small area (<1 km^2^). Among these ecosystems are tree nurseries (e.g., spruce, larch, pine) established by the *ministère des ressources naturelles-Québec* (MRNQ) for seed production to support reforestation programs. Fifteen years ago, the MRNQ converted part of the original agricultural area to tree plantations, leaving some parcels for agronomic production as well as unseeded lands that led to the emergence of a natural deciduous forest (MRNQ, pers. Commun.) For the purpose of this study, three adjacent areas with contrasting land-use types were sampled: maize monoculture area (stations A1, A2, A3), hybrid larch (*Larix marschlinsii Coaz*) plantation established by the MRNQ (stations M1, M2, M3) and the natural deciduous forest (stations F1, F2, F3). Three stations were identified in each ecosystem to collect composite soil samples (3 land-use types × 3 stations = 9 composite samples). Each composite soil sample consisted of six subsamples collected along a 2-m radius traced from a central point. The A-horizon was collected, from the soil surface to a depth of 10 cm as this zone comprises the highest CO uptake activity (King, 1999b). Surface litter in the forests and debris from the previous crop in the maize sites were removed before sampling. Samples were placed in plastic bags and transferred at 4°C within 2–4 h following their collection in the field. All samples were stored less than 3 months before laboratory analyses. A first soil survey was undertaken in April 2012. Soil of the maize monoculture was bare and unplowed, with a few crop residues remaining on the surface. Samples were used for CO uptake measurements, physicochemical analyses and *coxL* clone libraries. Sampling sites were visited for a second soil survey in November 2013. In contrast to the first survey, crop residues (i.e., senescent maize) resulting from plowing, were present at the maize monoculture sampling sites. Soil samples collected in 2013 were used for CO uptake and *coxL* qPCR assays.

### Soil physicochemical properties

Soil texture was determined with the hydrometer method and particle size distribution (Table 1) assigned soil samples to the silt loam textural class (Elghamry and Elashkar, 1962). Soil pH was analyzed in soil:water suspensions (1:2) and soil water content was measured using standard gravimetric method. Soil nutrients were analyzed in external service laboratories (INRS-Centre Eau, Terre et Environnement, QC, Canada). Phosphorus and potassium were analyzed by inductively coupled plasma optical emission spectrometry (ICP-AES) after acid extraction, while total carbon and total nitrogen soil content were determined using an elemental combustion system.

**Table 1:** **Physicochemical properties, potential CO uptake activity, and *coxL* richness in soil**.

Land-use type	Texture (sand/silt/clay)	C (%)	N (%)	P (mg kg^−1^)	K (mg kg^−1^)	pH	H_2_O (%)	CO (pmol g^−1^_(dw)_ h^−1^)	Simpson (*D*)	Shannon (*H*′)	Number of *coxL* clones
Maize	0.05/0.84/0.11	1.6 ± 0.2	0.09 ± 0.04	616 ± 24	471 ± 186	5.4 ± 0.4	17 ± 6	389 ± 52	0.01	4.0	24 ± 3
Larch	0.03/0.88/0.09	2.3 ± 0.2	0.14 ± 0.01	332 ± 17	382 ± 115	4.7 ± 0.2	38 ± 10	227 ± 221	0.01	4.0	31 ± 7
Deciduous	0.14/0.84/0.02	11 ± 4	0.59 ± 0.23	290 ± 95	174 ± 78	4.0 ± 0.3	59 ± 4	1962 ± 1117	0.03	3.4	37 ± 8

Average values (three sampling stations per land-use type) are represented with standard deviations.

### Microorganisms

*Mycobacterium smegmatis* (DSMZ 43756) and *Stappia kahanamokuae* (DSMZ 18969) were purchased at the Leibniz Institute DSMZ—German Collection of Microorganisms and Cell Cultures, while *Burkholderia xenovorans* LB400 was kindly provided by the laboratory of Dr. Michel Sylvestre (INRS-Institut Armand-Frappier). *M. smegmatis* and *B. xenovorans* were grown in PYE broth (Kimble et al., 1995) and Bacto Marine Broth (Difco 2216) was used for the growth of *S. kahanamokuae*. Cultures were incubated at 30°C under 200 rpm agitation. Triplicate cultures dedicated to CO uptake measurements (20 ml) were incubated in gastight 500 ml Wheaton^®^ glass bottles equipped with a rubber septum cap. Defined volume of CO gas mixture (508 ± 10 ppmv CO, GST-Welco, PA, USA) was injected to get ~3 ppmv in the static headspace after inoculation. Headspace samples were collected during the incubation period to measure CO oxidation activity (see below). Independent triplicate cultures were also prepared in parallel to monitor bacterial growth by optical density readings. The biomass of stationary phase cultures was quantified by agar plate enumeration using PYE and Bacto Marine agar media.

### CO uptake activity

CO uptake activity was measured using either soil samples [~50 g_(drybasis)_] or bacterial cultures. Soil samples were placed into 500 ml gastight Wheaton^®^ glass bottles with rubber septum caps. Diffusion limitation of the activity was negligible since preliminary experiments showed proportional CO uptake activity as a function of the amount of soil in the assay using 25, 50, 75, and 100 g soil samples (data not shown). Bottles containing soil samples were tightly closed and CO gas mixture (508 ± 10 ppmv CO GST-Welco, PA, USA) was injected to get ~1 ppmv initial concentration in the static headspace. Decrease of the CO mixing ratio was monitored as a function of time by analyzing aliquots (10 ml) of the headspace air in a Trace Analytical Reduced Gas Analyzer (ta3000R, Ametek Process Instruments^®^, DE, USA) as previously described (King, 1999a). Apparent first order CO uptake rate constants were obtained by integrating the logarithmic decrease of headspace CO mixing ratio. Measurements were performed using biologically independent triplicates and at least five CO concentration points were used for data integration. Experiments involving soil samples were accomplished over 20–60 min, depending on microbial activity. CO uptake activity in bacterial cultures was measured over a 7-day period. Cell-specific CO oxidation rate (pmol cfu^−1^ h^−1^) was calculated by normalizing the activity to biomass as determined by agar plate enumeration. Reproducibility of the CO analyses was assessed before each set of measurements by repeated analysis of certified CO standard gas (2.05 ± 0.10 ppmv, GST-Welco, PA, USA), and standard deviations were lower than 5%. No significant CO uptake was observed for blank experiments involving sterile media or empty glass bottles. Considering the occurrence of simultaneous CO production and consumption activities in nature and their dependence on temperature, moisture and solar radiations, rates of CO oxidation in soil presented in this study must be considered as potential CO uptake activities.

### *coxL* phylogenetic analysis and PCR detection assays

Sequences similar to *coxL* in *Mycobacterium smegmatis* (type I-CODH) and *Burkholderia xenovorans* (type I- and type II-CODH) were retrieved from the National Center for Biotechnology Information (NCBI) database (http://www.ncbi.nlm.nih.gov/) using the protein Basic Local Alignment Search Tool (Altschul et al., 1990). Nucleic acid sequences were imported in the software Mega (Tamura et al., 2007), translated *in silico*, and the amino acid sequences were aligned using Muscle (Edgar, 2004). Alignments were manually refined and functional amino acid sequence motifs of the active site distinguishing *coxL* sequences belonging to type I-CODH (AYXCSFR) from type II-CODH (AYRGAGR) were examined in order to validate specificity of the retrieved sequences (King and Weber, 2007). Phylogenetic tree of amino acid sequences translated from *coxL* gene sequences was constructed with maximum-likelihood algorithm. The alignment was used to identify consensus regions to design *coxL*-specific oligonucleotides. Three sets of degenerated primers were developed to detect and quantify presumptive CO-oxidizers in soil, so-called universal-*coxL*, type I-*coxL*, and δ-Proteobacteria-coxL assays (Table 2).

**Table 2:** **List of the primers designed in this study**.

Assays (*coxL*)	Primers	PCR-amplified fragment size (bp)
Universal (PCR)	uni820-forward:	800
	5′-GGBGGBGGYTTYGGCWMSAA-3′	
	uni1611-reverse:	
	5′-GTBKCRTGNCCCTGNCC-3′	
Type I (qPCR)	type I-1288-forward:	252
	5′-TSKKYACSGGCWSSTA-3′	
	type I-1540-reverse:	
	5′-TAYGAYWSSGGYRAYTA-3′	
δ-Proteobacteria (qPCR)	D967-forward:	291
	5′-TTCTWCKCYGAYGCVCARCC-3′	
	D1285-reverse:	
	5′-CBGAGTCGGDSASGAADCC-3′	

16S rRNA gene sequences were retrieved from the genome of 102 presumptive CO-oxidizing bacteria identified in the *coxL* database (Table S1). 16S sequences were classified into two databases: type I-*coxL* and type II-*coxL* groups, as a function of *coxL* gene harbored by the bacteria, and then aligned. Pairwise difference (*D*) matrices were computed to obtain the similarity scores *S* (*S* = 1 − *D*) of all possible combinations of 16S rRNA and *coxL* gene sequences for both databases. Comparisons between the percentages of similarity of all *coxL* pairs and the sequence similarities of the 16S rRNA genes of the same bacteria were performed by regression analysis (*n* = 820 and *n* = 1830 for type I and type II databases, respectively). This pairwise similarity score analysis has recently been utilized to establish a similarity score threshold value for nitrate/nitrous oxide reductases (Palmer et al., 2009), particulate methane monooxygenase (Degelmann et al., 2010), and hydrogenase (Constant et al., 2011b) gene sequences at the species level.

### DNA extraction and universal-*coxL* PCR

Soil DNA was extracted from an exact amount of soil (~500 mg) using the FastDNA Spin Kit (MP Biomedicals^®^, OH, USA) for soil according to the manufacturers protocol. DNA was eluted in 50 μL nuclease-free water. DNA samples were diluted (1:10, 1:100, and 1:500) before the PCR due to residual humic acids inhibition. All PCR mixtures consisted of 1× reaction buffer (15 mM MgCl_2_), 0.2 mM deoxynucleotide triphosphates, 10 μM of each primer (Table 2), 0.8 mg ml^−1^ bovine serum albumin, 1.25 U Fast-Taq polymerase (Feldan^®^, QC, Canada), 2 μl diluted DNA and nuclease-free water to obtain a final volume of 50 μL. A touchdown PCR protocol was used for the universal-*coxL* assay as follow: 95°C for 5 min, 16 cycles of “touchdown steps” denaturing at 95°C for 20 s, annealing temperature starting at 65°C decreasing 0.5°C in every cycle to reach a temperature of 55° (40 s at each cycle), and a elongation step of 72°C for 45 s, completed with a final set of 19 regular PCR cycles of 95°C for 20 s, 55°C for 40 s and 72°C during 45 s with a final extension of 72°C for 5 min.

### *coxL* gene libraries

One *coxL* gene library was derived from each sampling station, resulting in nine fully replicated libraries. Partial *coxL* gene sequences were PCR-amplified using the universal-*coxL* assay and cloned in pGEM-T^®^ Easy Vector cloning Kit (Promega, WI, USA). Recombinant colonies were selected, plasmid DNA extracted following standard procedure (Sambrook and Russell, 2001) and *coxL* inserts were PCR-amplified and sequenced using the Sanger's Method (Génome Québec Innovation Centre, McGill University, QC, Canada). In total, 279 clones were obtained. Clone sequences were aligned and *in silico* translated to verify the canonical signature of the active site characterizing type I and type II *coxL* sequences. The OTU representative sequences (0.90 similarity cut-off) obtained using the universal-*coxL* assay were deposited in the GenBank database with accession numbers KJ395119 to KJ395310. UniFrac distance matrix, reflecting the pairwise phylogenetic distance between the sequences retrieved from each sampling site was calculated to verify if land-use types have significantly different microbial communities (Lozupone and Knight, 2005).

### Type I- and δ-proteobacteria-*coxL* qPCR assays

Type I- and δ-Proteobacteria-*coxL* genes were PCR-amplified using the universal-*coxL* assay and plasmid DNA of clone 55M3 (accession number KJ395179) and genomic DNA of *Haliangium ochraceum* DSM 14365 as matrices, respectively. PCR products were concentrated and purified with standard commercial kits (E.Z.N.A. Cycle Pure Kit, Omega Bio-Tek^®^, GA, USA). Purified DNA was quantified with fluorescent DNA-binding dye (Quantifluor dsDNA, Promega, WI, USA). Standard curves for type I- and δ-Proteobacteria-*coxL* qPCR assays were obtained using serial dilutions of quantified DNA (10^1^–10^9^ copies μl^−1^). Reactions contained 1× Perfecta SYBR Green Fast Mix reaction buffer (Quanta Biosciences^®^, MD, USA), 15 μM of each primer (Table 2), 0.3 mg ml^−1^ bovine serum albumin, 5 μL diluted DNA (1:500) and nuclease-free water to obtain a final volume of 20 μL. Preliminary experiments with internal standard DNA spiked in soil DNA extracts (Deer et al., 2010; Decoste et al., 2011) were conducted and showed undistinguishable qPCR-signal recovery between the samples using 1:500 DNA dilutions. Furthermore, qPCR results from 1:500 to 1:1000 dilutions were undistinguishable, providing no significant incidence of PCR inhibitors on *coxL* abundance data (data not shown). Reactions were performed in the Rotor Gene 6000 (Corbett Life Science^®^, NSW, Australia) with the following conditions: 94°C for 5 min, 35 cycles of 94°C for 30 s, 51°C (Type I-*coxL*) or 56°C (δ-Proteobacteria-*coxL*) for 30 s, 68°C for 20 s (Type I-*coxL*) or 15 s (δ-Proteobacteria-*coxL*) with fluorescence acquisition following each 68°C step and a melting cycle with a ramp from 75 to 99°C, rising 0.2°C every 5 s. Replicate calibration curves were performed to verify the accuracy of the qPCR resulting in an efficiency of 0.70 (*R*^2^ = 0.98) and 0.73 (*R*^2^ = 0.96) for type I- and δ-Proteobacteria-*coxL* assays, respectively. Type I- and δ-Proteobacteria-*coxL* gene libraries were also performed to confirm the specificity of the assays. The resulting type I- and δ-Proteobacteria-*coxL* sequences with more than 200 pb length have been deposited in the GenBank database with accession numbers KJ567007 to KJ567022 and KJ567023 to KJ567040, respectively.

### Statistical analysis

Gene libraries were normalized to the sequencing effort of the smallest *coxL* library to avoid biases in comparative analyses introduced by the sampling depth. Using the software Mothur (Schloss et al., 2009), 24 *coxL* sequences were randomly selected from the nine libraries. The resulting sequences were grouped into operational taxonomic units (OTU) defined by a similarity level of 0.90. These files were used for diversity index calculation and statistical analysis. Redundancy analysis (RDA) was computed using the Vegan package (Dixon, 2003) implemented in R (R Development Core Team, 2008) according to the comprehensive procedure described by Borcard et al. (2011). RDA is a constrained analysis, used to extract structures of an observational dataset related to explanatory variables. In this study, RDA was considered to identify environmental variables influencing the structure of *coxL* gene profile in soil, in addition to highlight *coxL* sequences whose presence is related to elevated CO soil uptake activity. This test was preferred from canonical analysis due to the occurrence of several null values in the *coxL* data matrix. Soil variables (e.g., pH, carbon, nitrogen, water content, CO uptake activity) were standardized by subtracting individual values by the average and dividing them by the standard deviation. This transformation procedure resulted in centered data or *z*-scores, generating variables characterized by an average of zero and a standard deviation of 1. The Hellinger transformation was applied to *coxL* OTU frequency distribution before computing the distance matrix to avoid unduly relationships between explanatory variables and *coxL* composition supported by the high weight of rare species (Legendre and Gallagher, 2001). The most parsimonious constrained model to explain *coxL* composition was obtained by forward selection of the environmental variables (Blanchet et al., 2008) and permutation tests (*n* = 1000) were performed to assess the significance of the RDA. Pearson correlation analyses were conducted to identify environmental variables related to soil CO uptake activity. Analysis of variance with Bonferroni *post-hoc* statistical test was performed to compare CO uptake activity and abundance of *coxL* genes between the three land-use types (SigmaPlot 12^®^, Systat Software Inc., CA, USA).

## Results

### Soil properties and CO uptake activity

Triplicate composite soil samples (A-horizon) were collected in April 2012 to relate CO uptake activity to soil physicochemical properties and *coxL* diversity profiles. The highest carbon and nitrogen contents were detected in deciduous forest soil, while maize monoculture showed the maximum levels of potassium and phosphorus (Table 1). Distribution of the measured variables showed some level of co-linearity. Indeed, soil water content was positively related to K, P, and pH (Pearson correlation, *P* < 0.05) and inversely related to total carbon and nitrogen content (Pearson correlation, *P* < 0.01). Variations in soil physicochemical properties resulted in a broad range of CO uptake activities, from 45 pmol g^−1^_(dw)_ h^−1^ in larch plantation (station M2) to 3243 pmol g^−1^_(dw)_ h^−1^ in deciduous forest (station F3). The activity was positively correlated to carbon and nitrogen content in soil (Pearson correlation, *P* < 0.01), whereas no significant relationship was observed with the other variables. In accordance with total carbon and nitrogen profiles, CO uptake activity observed in deciduous forest soil was greater than in maize and larch plantations (ANOVA, *P* < 0.05), while soil samples collected from these two sites could not be distinguished based on their CO uptake activity. CO compensation concentration, reached when CO production and consumption rates are equivalent, was at the detection limit of the gas chromatographic system for the three ecosystem types (i.e., <25 ppbv), impairing estimation of the gross production and consumption rates of CO (Conrad, 1994).

### Detection of *coxL* genotypes

**Figure 1:**
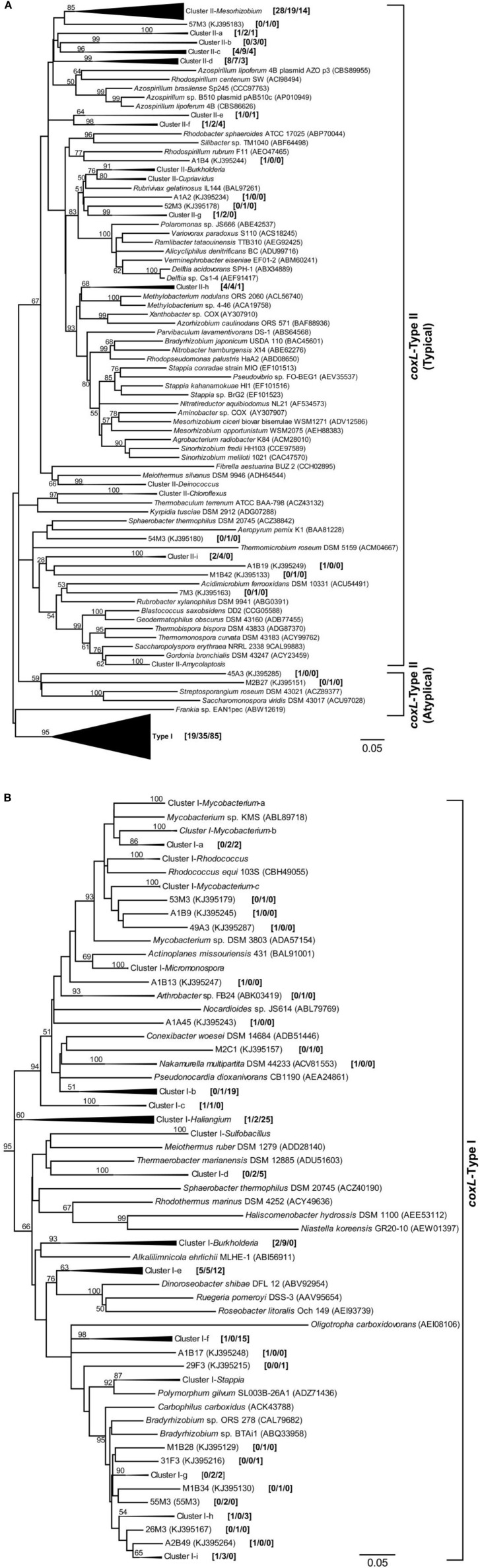
**Phylogenetic analysis of *coxL*-inferred amino acid sequences (313 residues) by the maximum-likelihood algorithm (model WAG+G)**. Global analysis including both type I- and type II-*coxL* sequences is shown **(A)** with a detailed view of *coxL*-type I phylogenetic group **(B)**. The analysis included sequences retrieved from public database along with the 192 OTUs identified in this study. The numbers in brackets show the number of *coxL* sequences from the nine clone libraries belonging to individual OTUs and clusters [maize/larch/deciduous]. The percentage of replicated trees in which the associated CoxL sequences clustered together in the bootstrap test (1000 replicates) are shown for nodes supported by ≥50% of the replicates. Prefixes of OTUs encompassing type I- and type II-*coxL* indicate land-use type as follow: A, maize monoculture; M, larch monoculture; and F, deciduous forest. Scale = number of substitutions per site.

**Figure 2:**
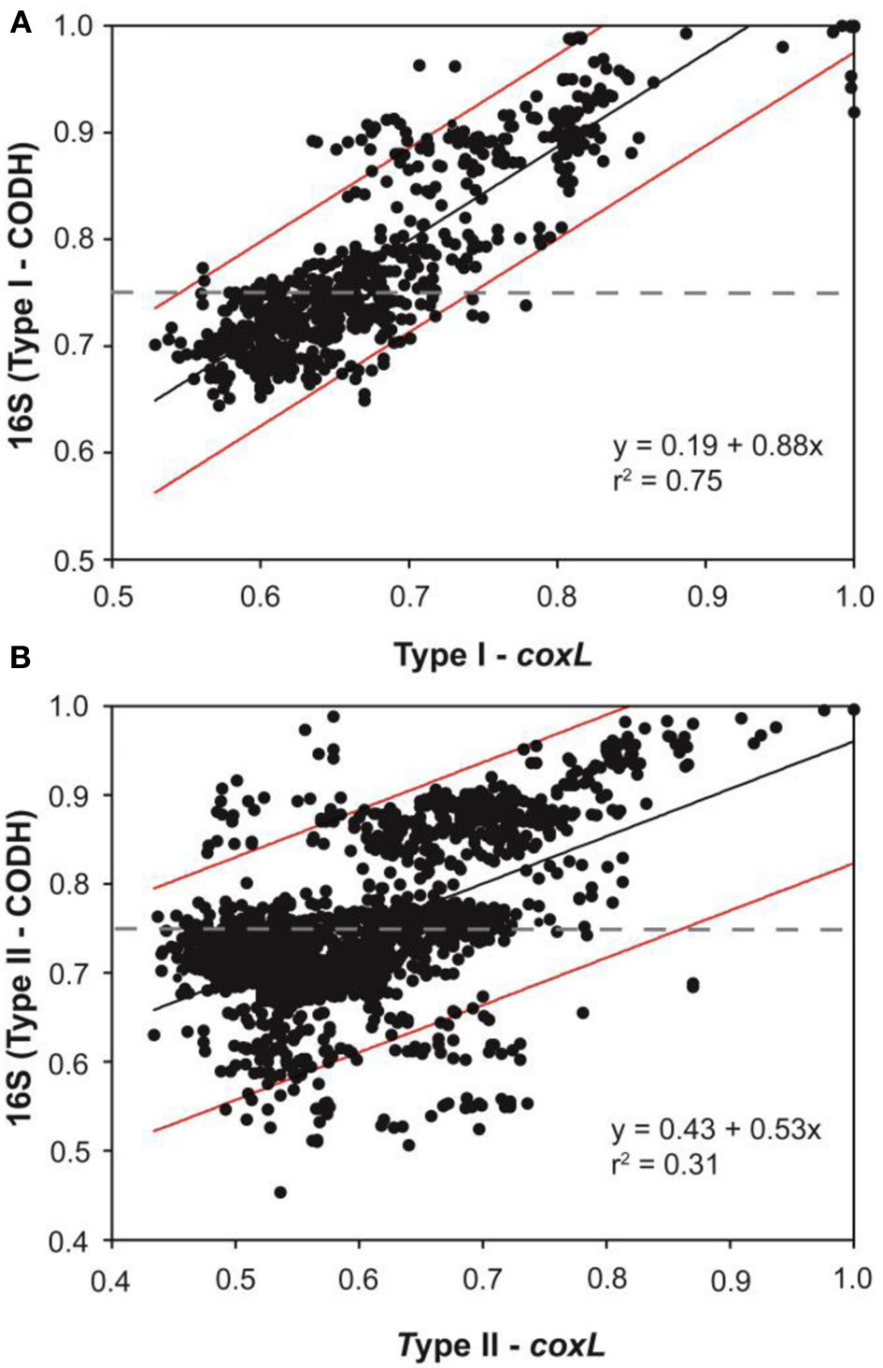
**Correlation between the pairwise sequence similarity scores of *coxL* and the 16S rRNA gene in presumptive CO-oxidizing bacteria belonging to (A) type I-CODH and (B) type II-CODH**. The black line represents the linear regression and red lines delineate the prediction interval of the model for the 95% confidence level.

An extensive phylogenetic analysis of *coxL* gene sequences was essential to get fundamental information regarding the evolution of functional type I-CODH and hypothetical type II-CODH, to optimize the universal-*coxL* PCR detection assay as well as to interpret gene libraries data. Putative *coxL* gene sequences were obtained from genome sequencing projects and CO-oxidizing bacteria exhibiting high affinity CO uptake activity (Figure 1). A parsimonious phylogenetic reconstruction of the type I-CODH group was obtained, while type II-*coxL* sequences were distributed in several clusters for which topology was poorly supported by bootstrap analysis (Figure 1A). Inspection of the conserved amino acid signature of the active site unveiled the occurrence of atypical motif in *Saccharomonospora viridis* and *Streptospotangium roseum* (Figure 1A). The PYRGAGR signature observed in these bacteria diverged from the canonical AYRGAGR motif of type II sequences. Pairwise sequence similarity scores of 16S rRNA and *coxL* genes were calculated to test whether standardization of the classification of *coxL* sequences is possible under “species-level” OTU and to assign environmental *coxL* sequences to taxonomic groups in phylogenetic analyses. The pairwise sequence similarity scores were correlated in bacteria possessing type I-*coxL* sequence, where the linear regression model (*n* = 820, *P* < 0.001) predicts a species-level similarity score threshold of 0.89 ± 0.04 (Figure 2A). For type II sequences, the regression model (*n* = 1830, *P* < 0.001) was associated to a species-level similarity score threshold of 1 ± 0.07 (Figure 2B), providing indication for different evolution histories for both types of CODH. Evidence of lateral transfer was noticed for type II-CODH. For instance, type II-*coxL* sequence detected in the aerobic hyperthermophilic Crenarchaeota *Aeropyrum pernix* was affiliated with that of a member of the Chloroflexi phylum (*Sphaerobacter thermophilus*), supporting potential lateral gene transfer event in the Archaea (Figure 1A). The extensive *coxL* database was utilized to optimize previous universal *coxL* PCR detection assay (Table S2).

**Figure 3:**
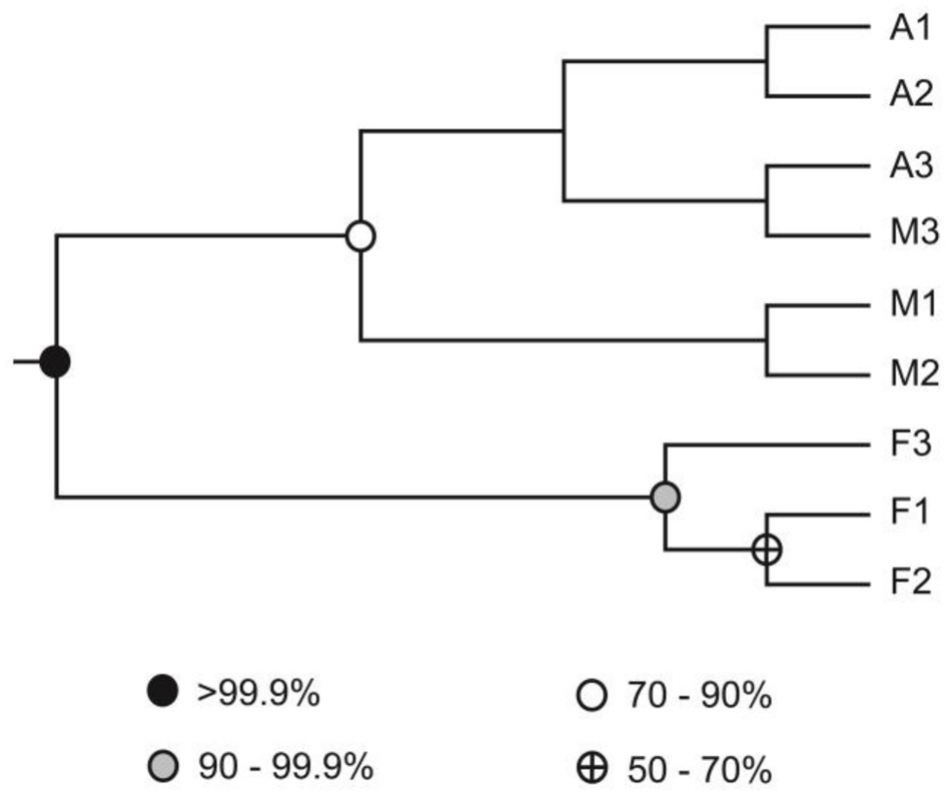
**UPGMA dendrogram of soil samples collected in deciduous forest (stations F1, F2, F3), maize monoculture (stations A1, A2, A3), and larch plantation (stations M1, M2, M3)**. The UPGMA was derived from the UniFrac distance matrix, reflecting the pairwise phylogenetic distance between the sequences retrieved from each sampling station. Nodes are filled as a function of the frequency at which they were found in Jackknife procedure keeping 75% sequences for the analysis.

**Figure 4:**
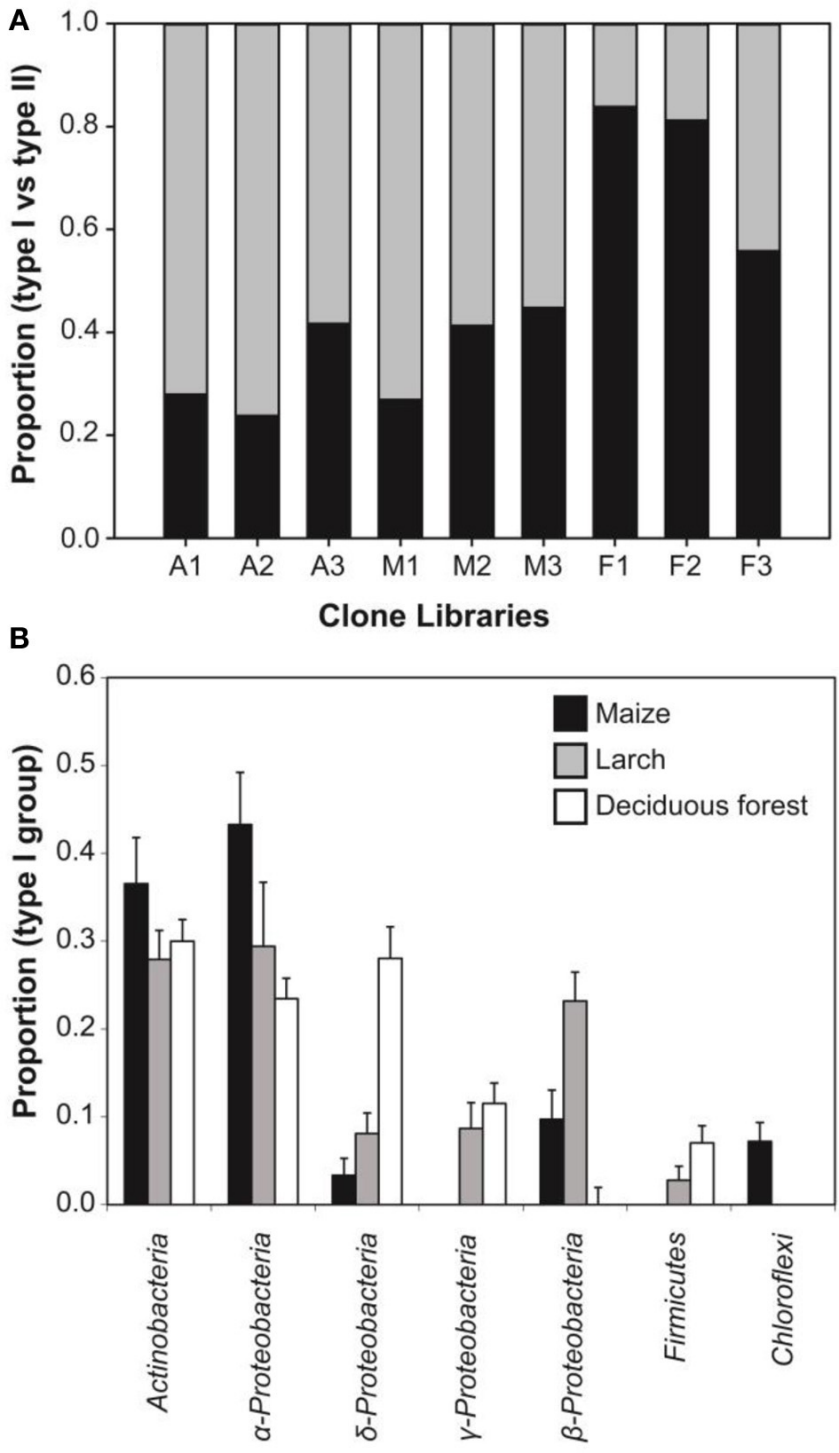
**(A)** Relative abundance of *coxL* clone sequences belonging to type I- (black) and type II-CODH (gray) groups in the nine clone libraries. **(B)** Percent composition (average ± standard error of three replicated clone libraries) of bacterial phyla represented by type I-*coxL* sequences detected in the three different ecosystems.

Genomic DNA was extracted from nine composite soil samples and *coxL* genes were PCR-amplified, cloned and sequenced. In total, 279 clones were derived from the maize (73), larch (93), and deciduous forest (113) samples. Sequences were classified into 192 different OTU using an arbitrary cut-off of 10% difference to accommodate both type I- and type II-*coxL* sequences. According to a rarefaction analysis, sampling effort was insufficient to cover the whole diversity of presumptive CO-oxidizing bacteria communities (data not shown). Comparison of the gene libraries thus are representative of the dominant members of this functional group in soil. Diversity metrics indicate lower richness of *coxL* sequences sampled in deciduous forest soil than the monocultures (Table 1). The lower value of the Simpson index in deciduous forest reflects dominance of the sampled community by a small number of OTU. UniFrac analysis of the nine *coxL* gene libraries was in accordance with the diversity metrics. Composition of deciduous forest *coxL* gene libraries differed significantly from maize and larch monocultures, while conversion of the agricultural field to larch monoculture 15 years ago did not influence the composition of dominant presumptive CO-oxidizing bacteria (Figure 3). The relative abundance of clones belonging to types I and II varied as a function of land-use type (Figure 4A). Type I-*coxL* sequences dominated deciduous forest soil, while maize and larch plantations displayed higher proportion of type II. Clone sequences belonging to type II were phylogenetically-distant from cultured representative bacteria. With the exception of the OTU A2B46, affiliated to *coxL* sequence from *Mesorhizobium loti* (73% similarity score, implying both sequences are derived from bacteria that could belong to two different phyla; Figure 2B), no type II sequence related to clusters comprising known CO-oxidizing bacteria was detected (Figure 1A). Phylotypes affiliated to the atypical *coxL* sequence of *S. viridis* and *S. roseum* were detected in the three ecosystem types, representing 0.7% of the analyzed clones. Most of the clones encompassing type I-*coxL* were comprised in *Actinobacteria* (16%), α-Proteobacteria (14%), and δ-Proteobacteria (10%) clusters. The proportion of clone sequences related to these phyla varied as a function of land-use type. For instance, 27% type I sequences detected in deciduous forest encompassed the δ-Proteobacteria cluster, while this group represented 6 and 10% in maize and larch monocultures (Figure 4B).

### Relationship between *coxL* gene sequences and environmental variables

**Figure 5:**
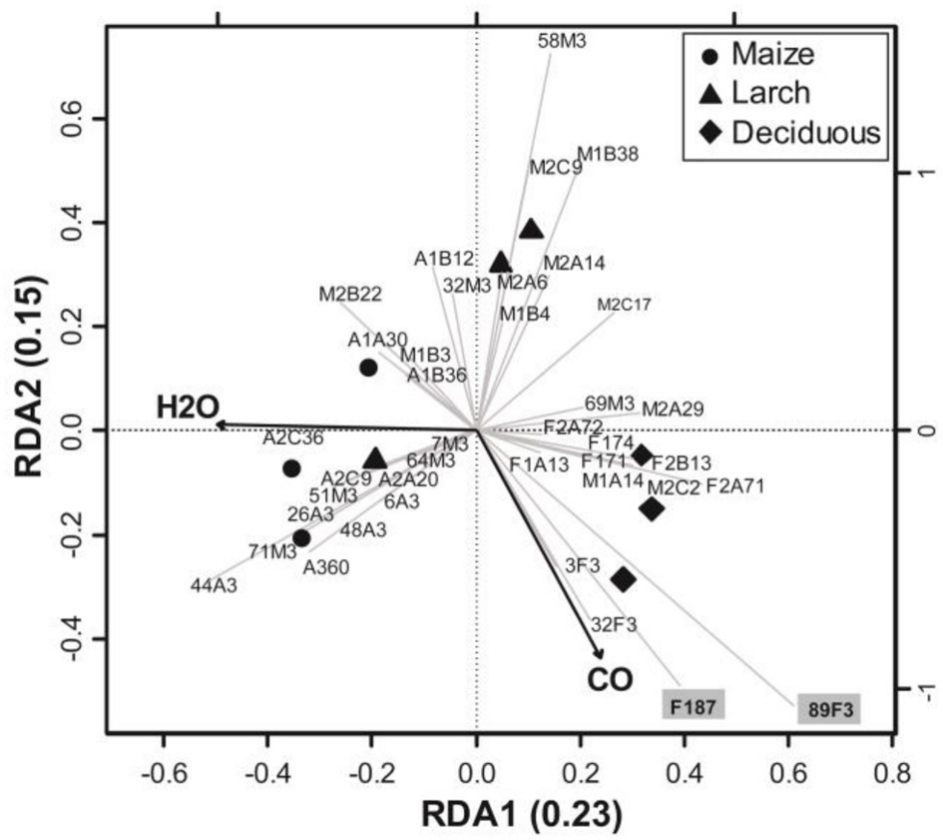
**Parsimonious RDA triplot of Hellinger-transformed *coxL* OTU frequency matrix explained by soil water content and CO uptake activity**. One OTU representative was kept for the analysis in the case of collinearly in *coxL* OTU profiles.

A RDA was performed to infer the relationship of *coxL* gene sequences with environmental variables (Figure 5). The most parsimonious model to explain variation of *coxL* sequences included soil CO uptake activity and water content. The other variables being redundant to CO uptake and soil moisture, their addition in the analysis increased the variance inflation factor unduly and they were therefore ignored in the analysis. The first two canonical axes explained 38% of the total variance of *coxL* OTU frequency distribution. Significance of the RDA was confirmed with 1000 permutations of *coxL* data matrix (*P* = 0.003). Soil water content played an important role for the dispersion of the samples along the first axis, while CO uptake activity discriminated the samples along the second. According to UniFrac analysis, axes clearly separated samples collected in deciduous forest from those originating from both monocultures (Figure 5). The occurrence of 12 OTU was related to higher CO uptake activities. Among them, 10 encompassed type I phylogenetic group (89F3, F187, 3F3 F2A71, M2C2, M1A14, F1A13, F2B13, F171, F2A72), while 2 belonged to type II (32F3, F174). Combined with the higher relative abundance of type I sequences detected in deciduous forest, this observation led us to consider that type I-*coxL* might be a better indicator of CO uptake activity in soil than type II sequences. OTUs 89F3 and F187 were related to deciduous forest samples characterized with the highest CO uptake activity (Figure 5). These sequences encompass the δ-Proteobacteria cluster, suggesting the relevance of this type I-*coxL* subgroup to predict CO oxidation activity in the soil samples. The obligate halophile myxobacterium *Haliangium ochraceum* isolated from coastal seaweed (Fudou et al., 2002) was the sole cultivated representative of the δ-Proteobacteria cluster, with no report on its CO uptake activity. We tested the CO uptake activity of *H. ochraceum* and confirmed its capability to scavenge atmospheric CO (Figure S1). This is the first demonstration of CO oxidation activity in the δ-Proteobacteria class.

### Linking CO uptake activity to the abundance of *coxL* sequences and theoretical populations of carboxydovores bacteria in soil

**Figure 6:**
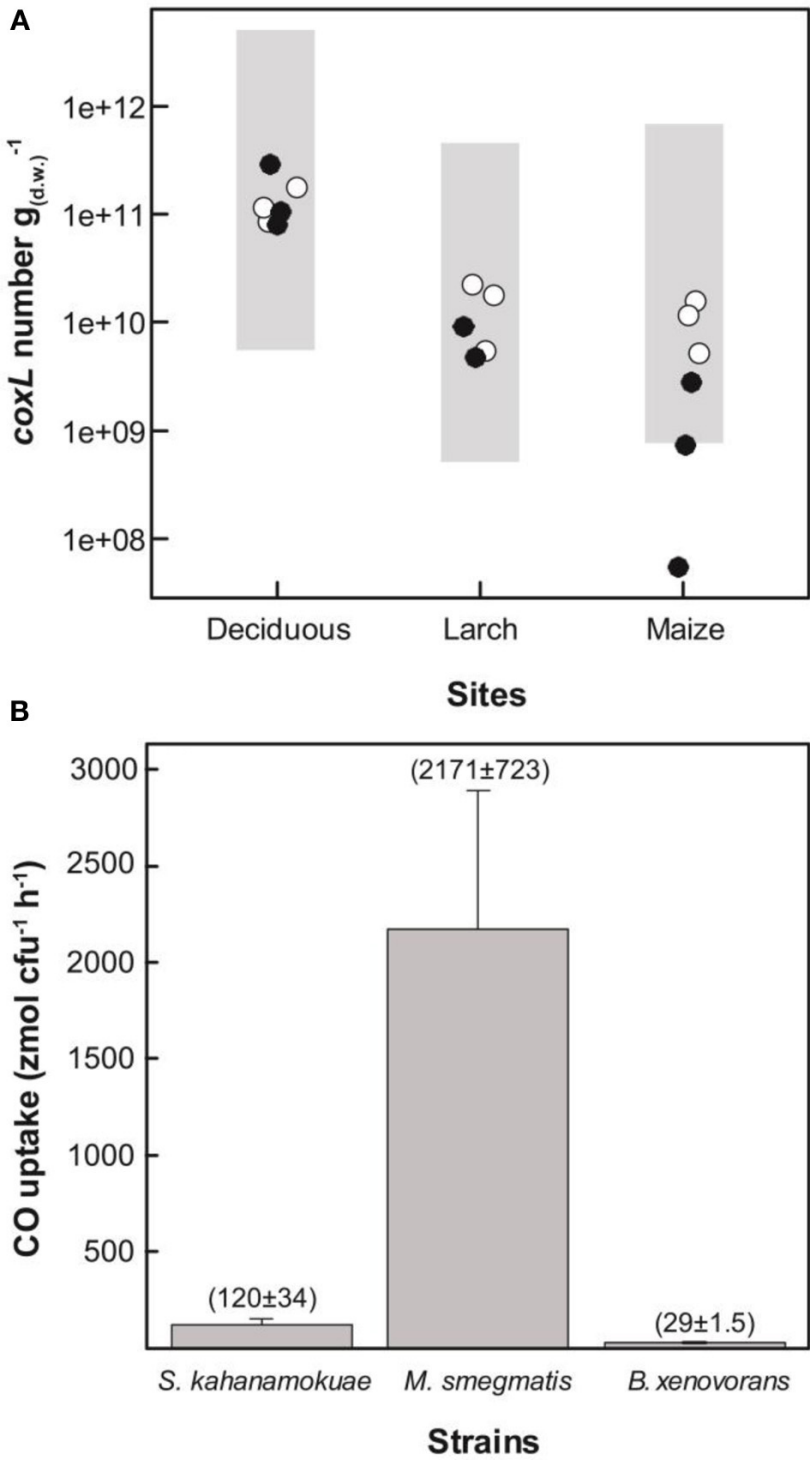
**(A)** Abundance of type I-*coxL* sequences (°) and δ-Proteobacteria-*coxL* sequences (•) in soil. Shaded areas represent the lower and upper limit of theoretical populations of CO-oxidizing bacteria. Average CO uptake rates measured in soil samples collected in 2013 (1.5, 1.0, and 11 nmol g^−1^_(dw)_ h^−1^ for maize, larch and deciduous forest, respectively), and cell-specific activity of *B. xenovorans* (upper limit of the population) and *M. smegmatis* (lower limit of the population) were used for the calculations (see panel **B**). **(B)** Specific CO uptake activity of selected CO-oxidizing bacteria.

**Figure 7:**
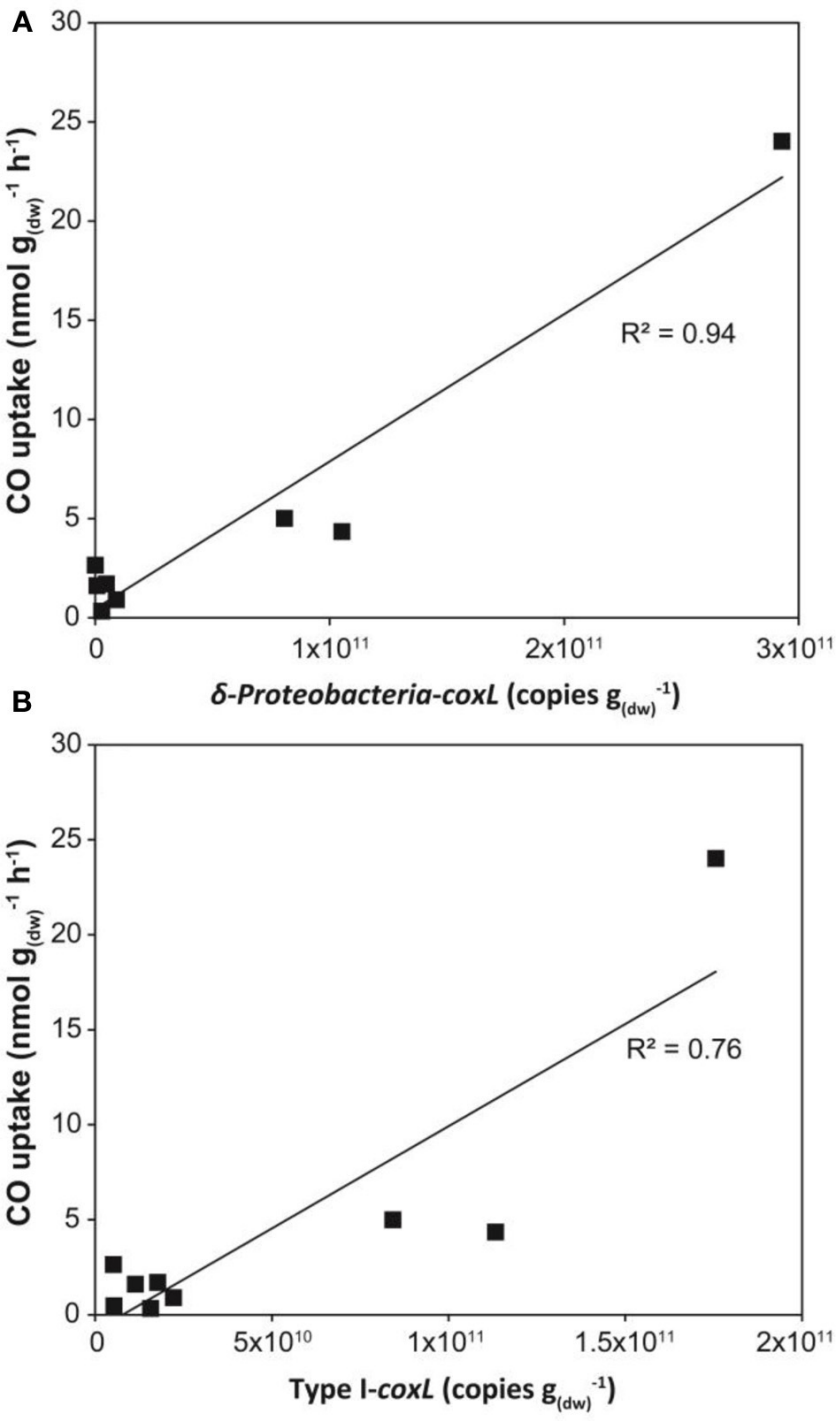
**Linear regression modeling dependence of CO uptake activity on the abundance of (A) δ-Proteobacteria-*coxL* and (B) type I-*coxL* gene number in soil assessed by qPCR**.

Gene libraries suggested that distribution of *coxL* sequences belonging to type I or the δ-Proteobacteria cluster reflect CO soil uptake activity. The analysis was however limited by insufficient sampling effort to cover the whole diversity of *coxL* sequences in soil as well as PCR and cloning bias. In order to challenge the results of clone libraries, the three sampling sites were visited for a second soil survey in 2013. CO uptake activity was measured and showed the same trend than the 2012 soil survey, with higher oxidation rates in deciduous forest than in monocultures, and total DNA was extracted for qPCR analyses. Degenerated oligonucleotides were designed to quantify *coxL* sequences belonging to type I group and δ-Proteobacteria subgroup. Optimization of a broad assay, specific type I-*coxL* sequences derived from public database and clone sequences obtained in this study was unsuccessful due to no or unspecific amplification signals induced by consensus degenerated primers (data not shown). As an alternative, oligonucleotides were designed based on the clone sequences only. Specificity of the assays was confirmed by *coxL* gene libraries (23 clones per assay) with 13 and 0% unspecific sequences for type I and δ-Proteobacteria, respectively. The abundance of type I-*coxL* varied between 10^9^ and 10^10^ genes g^−1^_(dw)_ in maize and larch monocultures and 10^10^–10^11^ genes g^−1^_(dw)_ in deciduous forest (Figure 6A). A similar trend was observed for the δ-Proteobacteria subgroup with an average of 10^9^ and 10^11^ genes g^−1^_(dw)_ for monocultures and deciduous forest, respectively, (Figure 6A). According to *coxL* gene libraries, type I-*coxL* sequences were more abundant in deciduous forest soil than both monocultures (*P* = 0.004). The abundance of δ-Proteobacteria *coxL* sequences and the relative proportion of this group was significantly higher in deciduous forest than maize monoculture (*P* = 0.01), while maize and larch monocultures pair as well as larch plantation and deciduous forest pair could not be distinguished based on the abundance of δ-Proteobacteria *coxL* sequences. Linear regression analyses showed that abundance of both *coxL* subgroups, as estimated by qPCR, was proportional to CO oxidation activity in soil (*P* < 0.003), but the relationships were largely driven by the contrasting properties of deciduous forest samples (Figure 7).

### Theoretical populations of carboxydovore bacteria in soil

Although differences in the efficiency of the qPCR reaction between standard and environmental DNA templates are expected due to the utilization of degenerated primers, the absolute quantification method remains a standard choice in environmental microbiology (Brankatschk et al., 2012). In order to verify the reliability of the qPCR estimates, three carboxydovore bacteria known to oxidize atmospheric CO and available in public microorganism culture collections were selected and characterized in term of cell-specific CO uptake activity. CO oxidation activity was detected at the onset of the stationary phase of the strains demonstrating a broad range in specific activities, from 29 to 2171 zmol cfu^−1^ h^−1^ (Figure 6B). This range in specific activities was used to calculate theoretical populations [*N*; cell g^−1^_(dw)_] of metabolically active carboxydovore bacteria necessary to explain the CO uptake activity measured in the second soil survey (Equation 1):

(1) $$N=\frac{CO_{Soil}}{CO_{Bacteria}}$$

where *CO*_*Soil*_ is the CO uptake activity measured in soil [pmol g^−1^_(dw)_ h^−1^] and *CO*_*Bacteria*_ is the CO oxidation rate in carboxydovore bacteria (pmol cfu^−1^ h^−1^; cfu = colony forming unit). The lowest and highest cell-specific CO oxidation activities (*CO*_*Bacteria*_) measured in *B. xenovorans* and *M. smegmatis* (Figure 6B) were utilized to calculate the upper and lower limits of *N*, respectively (Figure 6A, shaded areas). For this calculation, it is assumed that each bacterium harbors a single *coxL* operon and that one cfu corresponds to a unique viable cell. Considering the facts that some bacteria possess two *coxL* operons, the potential impact of cultivation conditions on CO uptake activity and the possibility that bacterial colonies arise from cell aggregates, this calculation provided a rough estimate of theoretical carboxydovore populations in soil. Nevertheless, with the exception of the abundance of δ-Proteobacteria *coxL* sequences in maize monoculture where qPCR data were below theoretical estimates, there was an agreement between theoretical populations and qPCR data (Figure 6A).

## Discussion

Land-use change exerts strong impact on biogeochemical cycles and soil microbial communities. These environmental pressures are especially marked for biogeochemical processes involving specific metabolisms, restricted to specialist microbes. For instance, afforestation of bog and grassland altered methanotrophic bacteria communities structure in soil, which was directly linked to an enhanced atmospheric methane soil uptake activity in land management experimental stations (Nazaries et al., 2013). Similarly, composition of N_2_-fixing and denitrifying microbial communities responded to soil physicochemical properties and land-use change, resulting in alteration of nitrous oxide fluxes following afforestation of pastures (Singh et al., 2011). The impact of land-use change on CO soil-to-air exchanges has been investigated in tropical and temperate climates. Exchanges measured in the field being influenced by temperature and soil water content, these investigations resulted in conflicting observations where agricultural areas represented either more important (King, 2000; King and Hungria, 2002; Pendall et al., 2010) or less important (Moxley and Smith, 1998) sinks for atmospheric CO than native forests. An unanswered question is whether variance of CO uptake rates observed in soil was due to change in CO-oxidizing bacteria populations in term of density, specific activity or diversity. Very few field studies combined CO uptake rate measurements with molecular survey of CO-oxidizing bacteria, impairing a clear assessment of the environmental control on their distribution and activity. One notable exception is an extensive survey of CO-oxidizing bacteria accomplished along volcanic deposits, demonstrating a gradient in community structure parallels to CO uptake activity (Weber and King, 2010a). Here, we seek to examine the occurrence of such microbial succession in three neighboring land-use types. The sampling strategy as well as replication of *coxL* gene libraries and physicochemical analyses were essential to assess the spatial distribution of CO-oxidizing bacteria in the surveyed ecosystems with confidence, in addition to identify the environmental factors best explaining their distribution (Prosser, 2010).

Although conversion of the maize plantation to larch monoculture 15 years ago resulted in significant changes in soil physicochemical properties (Table 1), it exerted no significant impact on CO uptake activity and *coxL* diversity. However, higher activity and distinct CO-oxidizing bacteria community structure were observed in deciduous forest soil, which emerged from fallow land without human intervention. CO uptake rates reported in Table 1 were in the same magnitude than the 0.3–50 nmol g^−1^_(dw)_ h^−1^ observed in temperate forest soil samples exposed to atmospheric CO (King, 1999a; Hardy and King, 2001). Soil carbon and nitrogen content were the best variables to explain variations of CO uptake activity. Even though such relationships have been observed in previous investigations, the mechanistic aspects of the simulation of CO uptake activity by soil nutrients have received little attention. In the case of nitrogen, correlation does not imply causation since previous investigations excluded nitrogen limitation of the activity. Indeed, ammonium soil amendments caused no influence on CO uptake rate, while nitrite addition resulted in transient inhibition of the activity (King, 1999a; Chan and Steudler, 2006). On the other hand, two main mechanisms have been proposed to explain how soil carbon content enhances CO uptake activity. Considering the fact that soil carbon content determines microbial biomass and soil respiration activity, it was first proposed that higher soil carbon content supported more abundant communities of CO-oxidizing bacteria (Inman et al., 1971; Moxley and Smith, 1998; King, 1999a). Secondly, an increase of the relative importance of *coxL* OTU to 16S rRNA gene OTU ratio as a function of soil organic carbon has been noticed, suggesting that soil carbon enhances diversity of CO-oxidizing bacteria relative to the whole microbial population in soil, resulting in an alteration in CO uptake activity (Weber and King, 2010a). Variation of the abundance and community structure of CO-oxidizing bacteria in response to carbon content in soil are likely induced by the occurrence of a larger pool of CO in organic rich soils, due to abiotic CO production reactions resulting from thermal- and UV irradiation-mediated soil organic matter decomposition (Conrad and Seiler, 1985; Sanhueza et al., 1998; Derendorp et al., 2011). Therefore, considering that low pH and high carbon content are known to promote CO production in soil (Moxley and Smith, 1998; King, 1999a), sampled deciduous forest may represent a more favorable niche for CO-oxidizing bacteria relative to maize and larch monocultures, resulting in the distribution of *coxL* sequences and CO uptake activities measured in this study.

The occurrence of *coxL* gene sequences belonging to type I and type II groups has been documented in forest, agricultural soils, and volcanic deposits. Investigations undertaken in volcanic deposits showed that type I-*coxL* diversity was correlated to soil respiration and CO uptake activity, while no significant correlation was found for type II-*coxL* sequences (Dunfield and King, 2005). This observation suggested that microbes belonging to type I and type II groups responded differently to environmental factors. Our analysis extends this proposal and unveils that type I-*coxL* sequences are better proxy for soil CO uptake activity than those encompassing the type II clade. The relative proportion of type I sequences was higher in deciduous forest soil demonstrating the highest CO uptake activity (Figure 4A), while maize and larch monocultures comprised higher proportion of *coxL* sequences belonging to type II-CODH. This was further supported by the qPCR assay, showing a direct link between type I-*coxL* gene number and CO uptake activity (Figure 7B). These observations, combined with experimental evidence obtained in previous investigations, question the physiological role of the hypothetical type II-CODH in bacteria and the relevance of this genetic marker for CO uptake activity. In contrast to type I-CODH, functional type II-CODH remains to be experimentally demonstrated. The best characterized type I-CODH is the enzyme from *Oligotropha carboxidovorans*, a carboxydotrophic bacterium unable to oxidize atmospheric CO due to its low affinity for this substrate (Conrad et al., 1981). Nevertheless, this classical CODH model unveiled critical features on genetic regulation and architecture of the active site (Santiago et al., 1999; Dobbek et al., 2002). Functional type II-CODH was proposed following the PCR-detection of type II *coxL* (and no detection of type I-*coxL*) in *Aminobacter* sp. COX, demonstrating high affinity CO-uptake activity (King, 2003a). The involvement of type II-CODH in CO oxidation reaction is however puzzling since the canonical cysteine residue accommodating the copper atom directly involved in the CO oxidation catalysis (Dobbek et al., 2002) is replaced by a glycine residue in these hypothetical enzymes. Furthermore, characterization of CO oxidation activity in marine *Roseobacter* spp. revealed that strains harboring type II-*coxL* only were not active (Cunliffe, 2010). Genetic investigations are mandatory to assess the physiological role of type II-CODH, but *coxL* sequences belonging to functional type I-CODH phylogenetic group appear more relevant to predict CO uptake activity in soil.

Previous soil survey for CO-oxidizing bacteria realized along volcanic deposits, agricultural areas and forests unveiled dominance of type I-*coxL* sequences belonging to α-, β-Proteobacteria, *Actinobacteria*, and *Chloroflexi*, with each group represented by strains for which CO uptake activity has been demonstrated (King and Weber, 2007). The α-Proteobacteria cluster comprises the model CO-oxidizing bacterium *Oligotropha carboxidovorans* able to grow using CO as only carbon source, as well as *Bradyrhizobium*, *Roseobacter*, *Ruegeria*, and *Stappia* representatives. Among these, *Stappia* isolates displayed a high affinity CO oxidation activity and thus, the ability to oxidize ambient and sub-ambient levels of CO (Weber and King, 2007). *Bradyrhizobium*, *Roseobacter*, and *Ruegeria* representatives were also shown to oxidize CO, but their affinity for CO has not been reported (King, 2003a; Tolli et al., 2006; Cunliffe, 2010). Oxidation of atmospheric CO in β-Proteobacteria mainly has been examined in *Burkholderia*. Metabolism of CO was unevenly distributed in this genus, with more prevalence in strains thriving in the rhizosphere, and CO oxidation rates were shown to be higher when heterotrophic growth substrates were limiting (King, 2003a; Weber and King, 2012). *Actinobacteria* were also shown to oxidize atmospheric CO, with mycobacterium as the most extensively studied group (King, 2003b; Song et al., 2010; Kim and Park, 2012). Finally, recent investigations demonstrated that capacity for CO uptake is a common trait among the *Ktedonobacteria*, in agreement with the detection of *coxL* sequence affiliated to this taxonomic group in cinder volcanic deposits (Weber and King, 2010a; King and King, 2014). These observations suggest that carboxydovore bacteria responsible for the measured CO uptake activity harbored the type I-*coxL* sequences detected in this study. Comparison of our analysis with previous investigations combining type I-*coxL* and CO uptake activity analysis suggests that environmental conditions select different groups of carboxydovores in soil. Indeed, analysis of a vegetation chronosequence in Hawaii highlighted an increase in β-Proteobacteria-*coxL* sequences in sites characterized by higher CO uptake activity, suggesting the importance of this taxonomic group for CO uptake activity (Weber and King, 2010a,b; King and King, 2014). It was proposed that CO-oxidizing *Burkholderia* spp. were favored with plant development, benefiting of root exudates for growth and elevated CO levels as energy source in the rhizosphere (King and Crosby, 2002; Weber and King, 2012). In this study, assignation of type I-*coxL* clone sequences to taxonomic groups showed a higher relative abundance of δ-Proteobacteria-*coxL* sequences in deciduous forest showing the maximal CO uptake activity (Figure 4B). Rare sequences affiliated to this cluster were detected in bare soil of volcanic deposits, with *H. ochraceum* as the closest cultivated relative (Weber and King, 2010a). We confirmed the ability of *H. ochraceum* to oxidize atmospheric CO, but the origin of detected δ-Proteobacteria-*coxL* sequences remains unknown as they share less than 75% similarity score with *H. ochraceum*. Although this carboxydovore is halophile, myxobacteria related to this genus are diverse and were detected in recent soil metagenomic surveys (Luo et al., 2014; Zhou et al., 2014). Myxobacteria are ubiquitous in soil and are characterized by the formation of fruiting bodies enclosing stress-resistant myxospores structures as well as their ability to metabolize recalcitrant carbon macromolecules and feed on prey microorganisms through exoenzyme secretion (Reichenbach, 1999; Dawid, 2000). As no other genome sequence of δ-Proteobacteria was shown to harbor *coxL* gene sequence in our genome data mining, isolation of more representatives within this taxonomic group deserves peculiar attention to investigate their contribution in the biogeochemical cycle of CO.

This article provides the first absolute abundance of type I-*coxL* sequences in soil. The abundance of type I sequences determined in this study was higher than the 10^8^ genes g^−1^ reported in volcanic deposits using a qPCR assay specific to *Burkholderia* (Weber and King, 2010b). Analysis of cell-specific CO oxidation activity in three selected carboxydovores was undertaken to assess reliability of the qPCR assays. There was a general agreement with the *coxL* gene numbers and theoretical populations of carboxydovores bacteria necessary to explain the CO uptake activity measured in soil. The broad range in theoretical population predictions was explained by variance in specific CO oxidation activities, varying from 29 to 2171 zmol cfu^−1^ h^−1^ among the tested isolates. Even though potential variability induced by the formation of cfu from cell aggregates cannot be excluded, similar variations were observed in previous comparison of CO uptake activity in carboxydovore bacteria. Indeed, activity measured in axenic cultures of *Stappia* sp. and *Stenotrophomonas* sp. varied between 6 and 100 μg CO mg^−1^_(protein)_ h^−1^ (King, 2003a). Substrate affinity, cell physiology and metabolic activity are potential explanations for such variability in specific activity estimates (Knief and Dunfield, 2005) and will need more attention in future investigations to address how CO shapes microbial communities in the environment. Gene libraries suggested a more pronounced enrichment of carboxydovores belonging to δ-Proteobacteria in deciduous forest relative to both monocultures, but a qPCR assay targeting this specific subpopulation contradicted this observation. Incongruence of theoretical populations of carboxydovores and the abundance of δ-Proteobacteria-*coxL* sequences estimated by qPCR in maize plantation highlights the fact that carboxydovores belonging to this class cannot be used as an universal proxy for CO uptake activity in soil, due to the response of CO-oxidizer to their environment resulting in the dominance of different taxonomic groups of carboxydovores in contrasting ecosystems (Dunfield and King, 2005; King et al., 2008; Weber and King, 2010a). Considering this observation, we recommend the broader qPCR assay we developed, targeting the whole type I-*coxL* cluster, to test the relevance of this molecular marker in predicting CO uptake activity in soil for future investigations. These additional efforts, including samples displaying a broad range of CO uptake activity, are necessary because the regression analysis reported in Figure 7B was largely supported by the high CO uptake activity and *coxL* abundance in deciduous forest.

In conclusion, this work demonstrates the non-random distribution of CO-oxidizing bacteria in contrasting ecosystems, with land-use as a driver of diversification for this functional group. We showed that composition and abundance of CO-oxidizing bacteria community structure reflected CO uptake activity in soil. The combination of two complementary methodological approaches applied to independent soil surveys provides strong support and confidence to these observations. In contrast to the functional type I-CODH, the physiological role of type II-CODH remains to be defined as their distribution does not appear directly linked to CO uptake activity in soil and CO-oxidizing bacteria. Although this study was limited to three ecosystems, the soil survey resulted in the development of a reliable qPCR assay targeting presumptive CO-oxidizing bacteria in soil. A more extensive survey, including more ecosystem types is however necessary to challenge this quantitative indicator to predict CO oxidation rate in the environment. Finally, in addition to describe diversity of carboxydovore bacteria, this work suggests this functional group represents a significant proportion of soil microbiota. For instance, density of high affinity H_2_-oxidizing bacteria responsible for 80% of the global loss of atmospheric H_2_ is typically between 10^6^ and 10^8^ cells g^−1^_(soil−dw)_, as estimated by qPCR targeting the gene *hhyL* specifying the large subunit of their high affinity hydrogenase (Constant et al., 2011b). These microorganisms compensate their low abundance by a much higher cell specific activity than carboxydovores, oxidizing H_2_ at a rate of 2–3 amol cfu^−1^ h^−1^ in some streptomycetes (Constant et al., 2011a). Because carboxydovores are abundant and taxonomically diverse, they should exert a significant impact on soil microbiota and biological processes. Future work thus should focus on the interactions of CO-oxidizing bacteria with microorganisms involved in other globally important biogeochemical functions. In addition to alter global budget of atmospheric CO, alteration of the distribution and activity of this functional group may have significant impacts on ecosystem services.

## Author contributions

Liliana Quiza and Isabelle Lalonde performed the experiments. Liliana Quiza, Isabelle Lalonde, and Claude Guertin participated to manuscript redaction. Claude Guertin introduced Philippe Constant to the sampling site. Philippe Constant designed the research and wrote the article.

## Conflict of interest statement

The authors declare that the research was conducted in the absence of any commercial or financial relationships that could be construed as a potential conflict of interest.
