# Supplementary material for: Land-use influences the distribution and activity of high affinity CO-oxidizing bacteria associated to type I-coxL genotype in soil
Source: Front Microbiol. 2014 Jun 12;5:271. doi: 10.3389/fmicb.2014.00271 (PMC4053681; doi:10.3389/fmicb.2014.00271)
Supplement: Supplementary file 1 [file Presentation1.PDF]

Supplementary Material

Impact of Land-Use on the Distribution and Potential Activity of CO-Oxidizing Bacteria in Soil

Liliana Quiza, Isabelle Lalonde, Claude Guertin, Philippe Constant*

Laboratory of Trace Gas Biogeochemistry, INRS-Institut Armand-Frappier, Laval, Québec, Canada

*** Correspondence:** Philippe Constant, Laboratory of Trace Gas Biogeochemistry, INRS-Institut Armand-Frappier, 531 boulevard des Prairies, Laval, Québec, H7V 1B7, Canada.
Philippe.Constant@iaf.inrs.ca

1. Supplementary Figures and Tables

In the next sections are provided the list of *coxL* sequences retrieved from public genome database and integrated into our extensive phylogenetic analysis (Table S1), specificity of the newly designed primers targeting *coxL* gene (Table S2) and the CO uptake activity measured in *Haliangium ochraceum* (Figure S1).

1.1 Supplementary Tables

Table S1. List of the included sequences in the extensive *coxL* database.

**A) Type I-*coxL***	
**Bacteria**	**Accession Number**
Mycobacterium smegmatis MC2 155	AFP37210
Mycobacterium sp. JLS	ABN96292
Mycobacterium sp. KMS	ABL89718
Mycobacterium sp. MCS	ABG06612
Mycobacterium sp. DSM3803	ADA57154
Mycobacterium vaccae	ABC48602
Mycobacterium phlei	ABC48603
Mycobacterium marinum M	ACC39118
Mycobacterium canettii	CCC42714
Mycobacterium ulcerans Agy99	ABL02876
Mycobacterium tuberculosis CTRI 2	AEM98823
Mycobacterium tuberculosis CCDC5180	AEJ49183
Mycobacterium tuberculosis CCDC5079	AEJ45540
Mycobacterium tuberculosis KZN 4207	AEB02509
Mycobacterium tuberculosis KZN 1435	ACT23405
Mycobacterium tuberculosis F11	ABR04721
Mycobacterium tuberculosis H37Ra	ABQ72100
Mycobacterium tuberculosis CDC1551	AAK44610
Mycobacterium tuberculosis RGTB327	AFE15356
Mycobacterium bovis BCG Mexico	AET17669

Mycobacterium bovis BCG Moreau RDJ	CCC62972
Mycobacterium africanum GM041182	CCC25447
Mycobacterium bovis BCG Tokyo 172	BAH24677
Mycobacterium bovis BCG Pasteur 1173P2	CAL70396
Rhodococcus opacus B4	BAH53526
Rhodococcus jostii RHA1	ABG97013
Rhodococcus equi 103S	CBH49055
Micromonospora sp. L5	ADU07549
Micromonospora aurantiaca ATCC27029	ADL45463
Actinoplanes missouriensis 431	BAL91001
Arthrobacter sp. FB24	ABK03419
Conexibacter woesei DSM14684	ABL79769
Nocardioides sp. JS614	ABL79769
Nakamurella multipartita DSM44233	ACV81553
Pseudonocardia dioxanivorans CB1190	AEA24861
Haliangium ochraceum DSM 14365	ACY14545
Alkalilimnicola ehrlichii MLHE 1	ABI56911
Meiothermus ruber DSM 1279	ADD28140
Sphaerobacter thermophilus DSM 20745	ACZ40190
Rhodothermus marinus SG0.5JP17 172	AEN74668
Rhodothermus marinus DSM 4252	ACY49636
Polymorphum gilvum SL003B 26A1	ADZ71436
Thermaerobacter marianensis DSM 12885	ADU51603
Bradyrhizobium sp. S23321	BAL79267
Bradyrhizobium japonicum USDA 6	BAL07124
Bradyrhizobium sp. ORS 278	CAL79682
Bradyrhizobium sp. BTAi1	ABQ33958
Burkholderia xenovorans LB400 chromosome 1	ABE30822
Sulfobacillus acidophilus DSM 10332	AEW04034
Sulfobacillus acidophilus TPY A	EJ40384
Dinoroseobacter shibae DFL 12	ABV92954
Ruegeria pomeroyi DSS 3	AAV95654
Oligotropha carboxidovorans OM5 pHCG3	AEI08106
Haliscomenobacter hydrossis DSM 1100	AEE53112

**B) Type II-*coxL***	
**Bacteria**	**Accession Number**
Burkholderia xenovorans LB400 chromosome 1	CP000270
Burkholderia sp. CCGE1002 chromosome 1	ADG14199
Burkholderia sp. CCGE1001	ADX53573
Burkholderia phytofirmans PsJN chromosome 1	ACD14794
Burkholderia sp. CCGE1003 chromosome 1	ADN56134
Burkholderia sp. YI23	AET90569
Variovorax paradoxus EPS	ADU35966
Burkholderia xenovorans LB400 chromosome 3	ABE35958
Streptosporangium roseum DSM 43021	ACZ89377
Thermomicrobium roseum DSM 5159	ACM04667
Saccharomonospora viridis DSM 43017	ACU97028
Saccharopolyspora erythraea NRRL 2338	CAL99883
Sphaerobacter thermophilus DSM 20745	ACZ38842

Thermobispora bispora DSM 43833	ADG87370
Aeropyrum pernix	ACL50613
Aeropyrum pernix K1	BAA81228.2
Ramlibacter tataouinensis TTB310	AEG92425
Kyrpidia tusciae DSM 2912	ADG07288
Methylobacterium nodulans ORS 2060	ACL56740
Methylobacterium sp. 4 46	ACA19758
Azospirillum lipoferum 4B	CBS86626
Azospirillum lipoferum 4B plasmid AZO p3	CBS89955
Azospirillum brasilense Sp245	CCC97763
Ralstonia eutropha H16	CAJ91588
Ralstonia eutropha JMP134	AAZ59806
Ralstonia solanacearum Po82	AEG69183
Ralstonia solanacearum PSI07	CBJ51264
Ralstonia solanacearum CFBP2957	CBJ43165
Ralstonia solanacearum CMR15	CBJ38719
Ralstonia solanacearum GMI1000	CAD15170
Ralstonia pickettii 12J	ACD26495
Ralstonia pickettii 12D	ACS62705
Meiothermus silvanus DSM 9946	ADH64544
Blastococcus saxobsidens DD2	CCG05588
Rubrobacter xylanophilus DSM 9941 A	BG03091
Rubrivivax gelatinosus IL144	BAL97261
Deinococcus gobiensis I 0	AFD26460
Deinococcus geothermalis DSM 11300	ABF46241
Geodermatophilus obscurus DSM 43160	ADB77455
Bradyrhizobium japonicum USDA 110	BAC45601
Polaromonas sp. JS666	ABE42537
Thermobaculum terrenum ATCC BAA 798 chromosome 2	ACZ43132
Acidimicrobium ferrooxidans DSM 10331	ACU54491
Verminephrobacter eiseniae EF01 2	ABM60241
Cupriavidus necator N 1 chromosome 1	AEI75824
Cupriavidus taiwanensis LMG 19424	CAP63049
Cupriavidus necator N 1 chromosome 2	AEI79735
Delftia acidovorans SPH 1	ABX34889
Delftia sp. Cs1 4	AEF91417
Chloroflexus sp. Y 400 fl	ACM55104
Chloroflexus aggregans DSM 9485	ACL23891
Chloroflexus aurantiacus J 10 fl	ABY36652
Azorhizobium caulinodans ORS 571	BAF88936
Alicycliphilus denitrificans K601	AEB84595
Alicycliphilus denitrificans BC	ADU99716
Mesorhizobium ciceri biovar biserrulae WSM1271	ADV12586
Thermomonospora curvata DSM 43183	ACY99762
Frankia sp. EAN1pec	ABW12619
Rhodopseudomonas palustris BisA53	ABE38261
Rhodopseudomonas palustris BisB5	ABE40936
Rhodopseudomonas palustris HaA2	ABD08650
Rhodopseudomonas palustris BisB18	ABD90051
Rhodopseudomonas palustris DX 1	ADU46385

Rhodopseudomonas palustris TIE 1	ACF03636
Rhodobacter sphaeroides KD131	ACM01053
Rhodobacter sphaeroides ATCC 17025	ABP70044
Rhodobacter sphaeroides 2.4	ABA79038
Rhodobacter sphaeroides ATCC 17029	ABN76633
Rhodospirillum rubrum F11	AEO47465
Rhodospirillum rubrum ATCC 11170	ABC21767
Rhodospirillum centenum SW	ACI98494
Pseudovibrio sp. FO BEG1	AEV35537
Mesorhizobium opportunistum WSM2075	AEH88383
Mesorhizobium loti MAFF303099	BAB54109
Parvibaculum lavamentivorans DS 1	ABS64568
Amycolatopsis mediterranei S699	AEK40389
Amycolatopsis mediterranei U32	ADJ43681
Gordonia bronchialis DSM 43247	ACY23459
Niastella koreensis GR20 10	AEW01397
Agrobacterium radiobacter K84	ACM28010
Sinorhizobium fredii HH103	CCE97589
Sinorhizobium meliloti SM11	AEH80406
Sinorhizobium meliloti AK83	AEG54752
Sinorhizobium meliloti BL225C	AEG05716
Sinorhizobium meliloti 1021	CAC47570
Silibacter sp. TM1040	ABF64498
Nitrobacter hamburgensis X14	ABE62276
Fibrella aestuarina BUZ 2	CCH02895
Roseobacter litoralis Och 149	AEI93739
Variovorax paradoxus S110	ACS18245

Table S2. Optimisation of the uni-*coxL* assay developed by King (2003) and verification of the specificity of the type I-*coxL* assay developed in this study. Original and newly designed primers are on the grey and white lines, respectively.

**Name**	**Target**	**Sequence (5' to 3')**	**Specificity with "X" mismatches**						
			**N[1]**	**1**	**2**	**3**	**4**	**5**	**6**
OMP-forward	Type I	GGCGGCTTYGGSAASAAGGT	53	13	7	2	0	0	0
BMS-forward	Type II	GGCGGCTTYGGSTCSAAGAT	90	19	9	10	4	2	1
uni820-forward	Type I & II	GGBGGBGGYTTYGGCWMSAA	143	19	1	0	0	0	0
O/B-reverse	Type I & II	YTCGAYGATCATCGGRTTGA	143	30	23	6	4	4	1
uni1611-reverse	Type I & II	GTBKCRTGNCCCTGNCC	143	20	4	1	0	1	0
OMP1288-forward	Type I	TSKKYACSGGCWSSTA	53	8	1	1	0	0	0
OMP1540-reverse	Type I	TARTYRCCSSWRTCRTA	53	8	0	0	0	0	0

[1]The "N" column reports the number of utilized sequences in the *coxL* database to verify primer specificity. The next columns report the number of sequences in the *coxL* database having 1, 2, 3, 4, 5 or 6 mismatches with the corresponding primer.

1.2 Supplementary Figure 1

Growth of *Haliangium ochraceum* DSM 14365 (purchased at the Leibniz Institute DSMZ – German Collection of Microorganisms and Cell Cultures) in liquid medium was not successful. We thus adapted the CO oxidation assay to biomass grown on agar media. Briefly, 500 ml gastight Wheaton® glass bottles with rubber septum caps were sterilized and aseptically filled with 50 ml VY/4-SWS Agar (DSMZ 985 medium). The pre-poured bottles were left to stand at 4ºC during one week, with the septum caps loosen but covered with aluminum foil, to allow background CO gas produced during medium sterilisation to evacuate the bottles before the experiments. The agar bottles were then inoculated with a dense inoculum of *H. ochraceum* harvested from a plate to ensure confluent bacterial growth in the bottles. Defined volume of CO gas mixture (508 ± 10 ppmv CO, GST-Welco, Pennsylvania, U.S.A.) was injected to get ~3 ppmv in the static headspace after inoculation and the bottles were incubated 9 days at 30°C. Decrease of the CO mixing ratio was monitored as a function of time by analyzing aliquots (10 ml) of the headspace air in a Trace Analytical Reduced Gas Analyzer (ta3000R, Ametek Process Instruments®, Delaware, U.S.A.). Although expression of cell-specific activity (zmol c.f.u.$^{-1}$ h^{-1}) was not possible, this approach was appropriate to confirm CO uptake activity in the strain (Figure S1).

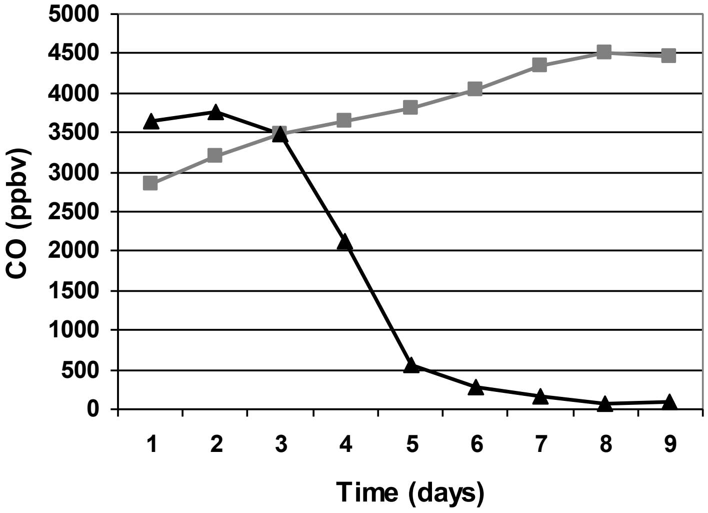

Figure S1. Representative CO oxidation activity in *H. ochraceum* (black triangle; ▲) in comparison to background emissions of CO in control experiment containing sterile medium (grey squares; ■). The lowest CO concentration measured in the headspace of *H. ochraceum* was 66 ppbv, confirming the ability of the strain to scavenge atmospheric CO (typical background level of CO in the atmosphere is ~120 ppbv).

2. References

King, G. M. 2003. Molecular and culture-based analyses of aerobic carbon monoxide oxidizer diversity. *Applied and Environmental Microbiology* 69:7257-7265.
